# The Effect of Hand Dimensions, Hand Shape and Some Anthropometric Characteristics on Handgrip Strength in Male Grip Athletes and Non-Athletes

**DOI:** 10.2478/v10078-011-0049-2

**Published:** 2011-10-04

**Authors:** Ali Asghar Fallahi, Ali Akbar Jadidian

**Affiliations:** 1 Department of exercise physiology, Faculty of Physical Education, Tehran, Iran; 2 Secretary of Talent Identification Committee of Handball Federation I.R.Iran; 3 Msc of corrective exercise. Level 1 anthropometry technician

**Keywords:** handgrip, hand dimensions, handgrip-related sports

## Abstract

It has been suggested that athletes with longer fingers and larger hand surfaces enjoy stronger grip power. Therefore, some researchers have examined a number of factors and anthropometric variables that explain this issue. To our knowledge, the data is scarce. Thus, the aim of this study was to investigate the effect of hand dimensions, hand shape and some anthropometric characteristics on handgrip strength in male grip athletes and non-athletes. 80 subjects aged between 19 and 29 participated in this study in two groups including: national and collegian grip athletes (n=40), and non-athletes (n=40). Body height and mass were measured to calculate body mass index. The shape of the dominant hand was drawn on a piece of paper with a thin marker so that finger spans, finger lengths, and perimeters of the hand could be measured. The hand shape was estimated as the ratio of the hand width to hand length. Handgrip strength was measured in the dominant and non-dominant hand using a standard dynamometer. Descriptive statistics were used for each variable and independent t test was used to analyze the differences between the two groups. The Pearson correlation coefficient test was used to evaluate the correlation between studied variables. Also, to predict important variables in handgrip strength, the linear trend was assessed using a linear regression analysis. There was a significant difference between the two groups in absolute handgrip strength (p<0.001) and handgrip/height ratio (p<0.001). The indices of body height, body mass, lean body mass and body fat content (p<0.001) were significantly greater in grip athletes. All hand variables except FS1-4 (p>0.05) were significantly different between the groups (p<0.001). After controlling body mass all hand anthropometric characteristics except thumb length (r=0.240, p= 0.135), hand shape (r=−0.029, p=0.858), middle finger length (r=0.305, p=0.056) and forearm circumference (r=0.162, p=0.319) significantly correlated with handgrip strength in grip athletes, but not in non-athletes, except for forearm circumference (r=0.406, p=0.010). The results showed that handgrip strength and some of the hand dimensions may be different in athletes who have handgrip movements with an object or opponent in comparison to non-athletes. Also, there was a significant positive correlation between handgrip strength and most of the hand dimensions in grip athletes. Therefore, these can be used in talent identification in handgrip-related sports and in clinical settings as well.

## Introduction

Handgrip strength is the maximal power of forceful voluntary flexion of all fingers under normal biokinetic conditions (Gandhi and Singh, 2008). Handgrip strength determines the muscular strength of an individual (Ling, et al., 2010; Fool, 2007). It is an important indication of general health and is regarded as one of the most reliable clinical methods for estimating strength (Nachon, et al., 2002; Hager-Ross and Schieber, 2000). Handgrip strength is important for catching and throwing the ball in different team sports. Also, when the fingers are longer and hand surface variables greater than required for grasping an object (ball), fingers will less widely spread, and grasping an object will become more efficient and less fatiguing (Nag and Desai, 2007).

With regard to grasping an object, ball or opponent, all sports can be divided into two groups: grasping or grip sports and non-grip sports. In grip sports, like basketball and handball, the longer the finger, the better the accuracy of the shot or throw. All shots and throws are finished with the wrist and fingers. It can be proposed that athletes with longer fingers and greater hand surface also have greater grip strength (Visnapuu and Jürimäe, 2007). In other grip sports such as wrestling, judo and rock climbing, hand strength can also be very important (Leyk et al., 2007; Grant et al., 2001; Watts et al., 2003). Handgrip strength is also important in determining the efficacy of different treatment strategies of hand and in hand rehabilitation (Gandhi and Singh, 2010). The handgrip measurement may be used in research, as follow-up of patients with neuromuscular disease (Wiles et al., 1990), as a predictor of all-cause mortality (Ling et al., 2010), as the functional index of nutritional status, for predicting the extent of complications following surgical intervention (Wang et al., 2010), and also in sport talent identification (Clerke et al., 2005).

Handgrip strength is affected by a number of factors that have been investigated. According to research, handgrip strength has a positive relationship with body height, body weight, body mass index, hand length, body surface area, arm and calf circumferences, skin folds, fat free mass, physical activity, hip waist ratio, etc (Gandhi and Singh, 2008; 2010). But, to our knowledge, hand anthropometric characteristics have not yet been investigated adequately. Handgrip strength has been investigated frequently. Some researchers have investigated handgrip strength in children and adolescents (Gandhi et al., 2010), while other studies have considered differences between the dominant and non-dominant hand. In recent studies, some groups of hand anthropometric variables were measured including: 5 finger spans, 5 finger lengths, 5 perimeters (Visnapuu and Jürimäe, 2007) and shape (Clerke et al., 2005) of the hand. Hand shape has been defined in various ways, but often as simply as the hand width to hand length ratio (W/L ratio). It seems that the differences of these parameters in athletes have not been indicated yet, and the information about these parameters is scarce. In fact, we hypothesized that grip athletes with specific hand anthropometric characteristics have different handgrip strengths when compared to non-athletes. Therefore, in the current study, we investigated the effect of hand dimensions, hand shape and some anthropometric characteristics on handgrip strength in male grip athletes and non-athletes.

## Material and Methods

### Participants

Totally, 80 subjects aged between 19 and 29 participated in this study in two groups including: handgrip-related athletes (n=40), and non-athletes (n=40). Handgrip-related athletes included 14 national basketball players, 10 collegian handball players, 7 collegian volleyball players, and 9 collegian wrestlers. National collegian basketball players were in conditioning camp for international championship of basketball in Filipina (2008) for three months. Collegian athletes, handball and volleyball players, and wrestlers were practicing in a conditioning camp for the 9^th^ national championships. All athletes trained with the frequency as follows: national basketball players, 10–12 sessions per week and approximately 18–20 hours; collegian handball players, volleyball players, football players, and wrestlers, 4–5 sessions per week, approximately 6–8.5 hours. Non-athletes did not participate in any sports.

### Anthropometric measurements

Body height (to nearest 0.1 cm) and body mass (to nearest 0.05 kg, komoshita seikosho, Yagam Japan) were measured and body mass index (BMI) was calculated as the body mass per (height)^2^ in kg/m^2^ as the general anthropometric variables. Body fat content was evaluated by measuring eight skin folds using level 1 Anthro kit and lean body mass was calculated. Measurement of anthropometrical variables of hand was a new original method reported by Visnapuu and Jürimäe (2007). First of all, the subjects were informed about the procedure of the study; then, they were asked to be seated comfortably and instructed to spread and stretch out their dominant hand and place it on a piece of paper located on the table. The outlines of the dominant hand were drawn by one examiner for all subjects. The contour of the hand was drawn with maximal active voluntary adduction of thumb and other fingers. Then three groups of hand anthropometric variables were measured: 5 finger spans, 5 finger lengths, and 5 perimeters of the hand.

**Figure 1:**
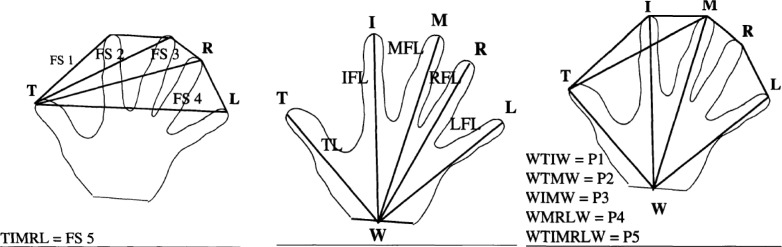
Outlines of anthropometric parameters and dimensions of hand: finger spans (FS1, FS2, FS3, FS4 and FS5), finger lengths (TL, IFL, MFL, RFL, LFL), and 5 perimeters (P1, P2, P3, P4, P5) (Visnapuu and Jürimäe, 2007)

Finger spans (FS1, FS2, FS3, FS4 and FS5), finger lengths (TL, IFL, MFL, RFL, LFL), and 5 perimeters (P1, P2, P3, P4, P5) of the hand (Figure 1) were measured by a standard 300-mm metal ruler (Visnapuu and Jürimäe, 2007).

Hand shape has been defined in various ways, but often as simply as the hand width to hand length ratio (W/L ratio).

Hand length of the dominant hand (the distance from the tip of the middle finger to the midline of the distal wrist crease when the forearm and hand are supinated on a table) (Okunribido, 2000), palm length (the distance between the midline of the distal wrist crease and the base of middle finger), F3 length (the middle finger length), forearm length (the joint line of proximal head to the styloid process), forearm circumference (the greatest circumference of forearm), wrist circumference (the circumference of wrist at wrist crease), and hand width (the distance between the radial side of the second metacarpal joint to the ulnar side of the fifth metacarpal joint) (Pheasant, 1996) were measured with anthro kit L1 and were recorded to the nearest millimeter. Handgrip strength was measured in the dominant and non-dominant hand with a standard dynamometer (DM-100s Yagami Japan).

### Statistical analysis

Our results were expressed as mean ± SD; therefore, descriptive statistics (mean and standard deviation) were calculated for each variable. Independent *t* test was used to compare the mean of variables in the two groups. Pearsoncorrelation coefficient test was used to evaluate the correlation between variables. Also, to predict important variables in handgrip strength, linear trend was assessed using linear regression analysis. The analyses were conducted using the SPSS version 16. Results were considered to be significant if their associated P-values were less than 0.05.

## Results

Table 1 illustrates the mean and standard deviation of general body and handgrip strength with the result of independent *t* test. Body height (p<0.001), body mass (p<0.001), lean body mass (p<0.001) and body fat content (p<0.001) were significantly greater in grip athletes in comparison with non-athletes. There was a significant difference between the two groups in absolute handgrip strength (p<0.001) and handgrip/height ratio (p<0.001), but not in handgrip/weight ratio (p<0.151). According to Table 2, there was no significant difference between the groups in finger spans 1–4 (p<0.05), except for FS5 (p<0.027); however, in 5 finger lengths (TL: p<0.001, IFL: p<0.001, MFL: p<0.00, RFL: p<0.001, LFL: p<0.001), and 5 perimeters (P1: p<0.001, P2: p<0.001, P3: p<0.001, P4: p<0.001, P5:p<0.013), there was a significant difference between the groups. In addition, hand length (p=0.002), palm width (p<0.001), F3 length (p<0.001), forearm length (p=0.013), forearm circumference (p<0.001) and wrist circumference (p<0.001) were significantly different between the groups. Hand shape and palm length were not significantly different between the groups (p>0.05).

The relationship between handgrip strength and baseline characteristics of athletes and non-athletes as well as handgrip strength and hand-specific anthropometric variables are summarized in Table 3 and 4. In athletes, body height (r=0.603, p<0.001), body mass (r=0.516, p=0.001), and lean body mass (r=0.536, p<0.001), but not body fat (r=0.0,79, p=0.630), as well as BMI (r=0.061, p=0.704) and in non-athletes, body mass (r=0.420, p=0.007), lean body mass (r=0.469, p=0.002) but not body height (r=0.230, p=0.153), body fat (r=0.420, p=0.428), and BMI (r=0.360, p=0.22) had a positive, significant correlation with handgrip strength. After controlling body mass, all hand anthropometric characteristics except thumb length (r=0.240, p= 0.135), hand shape (r=−0.029 p=0.858), middle finger length (r=0.305, p=0.056) and forearm circumference (r=0.162, p=0.319) significantly correlated with handgrip strength in grip athletes, but not in non-athletes, except for forearm circumference (r=0.406, p=0.010). Table 5 summarizes stepwise multiple regressions in which handgrip strength was the dependent variable and finger span, finger length, or finger perimeters were the independent variables.

## Discussion

The present study was conducted to investigate the effect of hand dimensions, hand shape and some anthropometric characteristics on handgrip strength in male grip athletes and non-athletes.

The major conclusion drawn from this study was that handgrip strength was significantly different between handgrip-related athletes and non-athletes. Also, approximately all hand anthropometric characteristics of grip athletes significantly correlated with handgrip strength, which indicates that these variables may have a positive effect on handgrip strength. There was no significant difference in hand shape, palm length and finger spans FS1, FS2, FS3, FS4, but not FS5, between the two groups. General body anthropometric characteristics (body height, body mass, lean body mass, and body fat content) were significantly different between the groups. Other variables, especially palm width, middle finger length, forearm circumference and wrist circumference were significantly different between the groups. The results showed that handgrip strength of athletes is greater than that of non-athletes. To our knowledge, there are only few studies that address this topic. This finding indicates that specific training of these sports may influence handgrip strength. Hand dimensions may influence handgrip strength and these athletes have biomechanical advantages.

Ruiz et al. (2006) showed that hand span influences optimal grip span in male and female teenagers. For males, the optimal grip span can be derived by the equation y = x/7.2 + 3.1 cm, and for females by the equation y = x/4 + 1.1 cm. In other words, it seems that finger spans are effective in handgrip strength. Some previous studies have suggested that the effect of finger length and finger variables on handgrip strength is more than that of finger spans so that finger spans have a small influence on handgrip strength (Visnapuu and Jürimäe, 2007). Results of the present study showed that finger spans, except for FS5, are not different between handgrip-related athletes and non-athletes, which require more research. Finger spans had a significant positive correlation with handgrip strength and FS5 is a good predictor of handgrip strength in grip athletes. These results can confirm the effect of finger spans on handgrip strength, specific athletic training, and performance in grip athletes; thus, these factors can be used in talent identification.

Also, results showed that palm length is not significantly different between the groups, but middle finger length is. This may be because of biomechanical effects of these hand parts, and it could be concluded that finger lengths may be more significant in handgrip strength than palm length.

It is especially necessary to measure finger length and perimeters of the hand for practical reasons. In a study, researchers have investigated the influence of hand dimensions on handgrip strength. Hager-ross and Schieber (2000), investigating children at different ages, confirmed that hand length (the distance from wrist joint to the tip of middle finger) is an important variable for handgrip strength. Studies of Nicolay and Walker (2005) showed that there is a significant but low correlation between finger length and handgrip strength in college students. Visnapuu and Jürimäe (2007) indicated that hand perimeters are the most important hand anthropometric variables in relation to handgrip strength. In the present study, all finger lengths and perimeters of the hand were significantly different (p<0.001) between the groups (Table 2) and had a significant correlation with handgrip strength (Table 4). These results indicate that in those sports where a hand is an important tool, training can be influenced by some anthropometric variables and hand dimensions as well as general anthropometric parameters that are related to maximal handgrip strength. In athletes who have handgrip actions, with object or opponent, some of the hand dimensions and parameters may be different in comparison with non-athletes and the other athletes who have no handgrip actions. This can be a base for research and for coaches in selecting athletes, or in clinical centers for more research in malformation of the hand.

It has been reported that throwing velocity is one of the most important factors for scoring in team handball (Marques et al., 2007). Tillaar and Ettema (2004) reported a positive correlation between isometric handgrip strength and ball-throwing velocity for female, as well as male team handball players. In this study, both before and after controlling body mass, we found that anthropometric parameters and dimensions of the hand including: finger spans (FS1, FS2, FS3, FS4 and FS5), finger lengths (IFL, MFL, RFL, and LFL), and 5 perimeters (P1, P2, P3, P4, and P5) have a high positive correlation with handgrip strength of the dominant hand. Hand shape did not correlate with handgrip strength in both groups, so it may not be significant in handgrip strength. Hand shape, especially in this method, may not be a useful variable for comparing athletes, who have griping tasks, and non-athletes.

After controlling body mass, hand length, palm width, forearm length and wrist circumference significantly correlated with handgrip strength in grip athletes. But thumb length, palm length, MF length, and forearm circumference did not show a significant correlation with handgrip strength. Forearm circumference significantly correlated with handgrip strength in non-athletes. This shows that in forearm circumference, the effect of body mass on handgrip strength is greater in grip athletes; thus, it did not significantly correlate with handgrip strength in the group of grip athletes.

According to Table 5, FS5 in athletes, IfL in the two groups, P5 in athletes and P4 in non-athletes between all finger spans, finger lengths and hand perimeters are predictors of hand grip strength. Therefore, these parameters and dimensions may be useful in talent identification and coaching in handball and similar sports.

Recently, Statkeviciene et al. (2010) reported that taller, fatter (and thus more buoyant) young individuals with smaller body-segment girths seem to enjoy their somatotype in the process of learning to swim freestyle, backstroke, and breaststroke. These hand anthropometrics and dimensions may also be related to the learning of grip-related sports, but this needs further research.

## Conclusions

In summary, we found that some hand dimensions and anthropometric parameters are different in the grip sports and non athletes, but we cannot exactly determine whether specific sport activities affect these differences or the inherent characteristics of athletes lead them to these sports. Also the handgrip strength of handgrip-related athletes was more than that of non-athletes. This may be because of hand anthropometric parameters and hand dimensions. In fact, good positive correlation between handgrip strengths and anthropometric characteristics of hand in grip athletes showed the effect of hand dimensions and anthropometry on handgrip strength in athletes who use their hands for grasping a ball or opponent. In addition, some of the hand dimensions (FS5, IFL and P5) in athletes may be good predictors of handgrip strength. Thus, these findings may be useful in the process of sports talent identification in grip sports such as handball, basketball, volleyball and baseball, as well as in other sports such as wrestling, judo, rock climbing and rowing.

## Figures and Tables

**Table 1:** Baseline characteristics and handgrip strength of subjects and P value of the t test

Hand variables	athletes (n=40)	Non-athletes (n=40)	P value
Age	23.39(2.69)	24.82(2.22)	0.037^*^
Body Height (cm)	1.82(10.12) 177.04(6.90)		0.001^**^
Body mass (kg)	85.25(11.18)	73.35(10.23)	0.001^**^
Lean body mass (kg)	77.7(9.82)	65.55(7.61)	0.001^**^
Body fat (%)	8.71(2.04)	10.31(3.05)	0.007^*^
BMI (kg x m^−1^)	24.42(1.88)	23.36(2.77)	0.021^*^
Handgrip strength			
DH (kg)	48.15(7.98)	39.70(5.88)	0.001^**^
NDH (kg)	45.64(7.21)	37.78(5.72)	0.001^**^
Handgrip-height ratio	0.257(0.03)	0.224(0.03)	0.001^**^
Handgrip-weight ratio	0.568 (0.08)	0.547(0.08)	0.269

Definition: BMI; body mass index. DH: dominant hand. NDH: non dominant

**Table 2:** Mean and standard deviation of specific and anthropometrical parameters of hand and P value of t test between groups (^*^; significant ^*^.α<0.05, ^**^ .α<0.001)

Hand Variable	Athletes (n=40)	Non-athletes (n=40)	*P* value
FS1	13.76±1.54	13.25±1.83	0.162
FS2	18.58±1.82	17.87±1.80	0.083
FS3	20.78±1.97	20.04±1.75	0.073
FS4	22.52±2.44	21.88±1.71	0.181
FS5	30.68±2.88	29.29±2.42	0.021^*^
TL	14.93±0.97	14.36±0.71	0.003^*^
IFL	19.58±1.23	18.72±0.99	0.001^*^
MFL	20.21±1.35	19.33±0.95	0.001^*^
RFL	19.15±1.32	18.30±0.95	0.002^*^
LFL	16.51±1.13	15.87±0.95	0.007^*^
P1	48.28±3.23	46.36±3.01	0.007^*^
P2	53.79±3.52	51.57±2.97	0.003^*^
P3	45.84±2.91	43.87±2.27	0.001^*^
P4	47.56±3.32	45.45±2.50	0.002^*^
P5	61.76±4.96	59.51±3.49	0.021^*^
Hand shape	0.43±0.02	0.43±0.01	0.386
Hand length	21.24±1.47	20.38±0.86	0.002^*^
Palm length	12.23±1.04	11.88±0.59	0.073
Palm width	9.21±0.44	8.77±0.33	0.001^**^
MF length	9.01±0.54	8.48±0.51	0.001^**^
Forearm length	26.86±1.84	25.76±2.05	0.013^*^
Forearm C	28.92±1.49	26.66±1.58	0.001^**^
Wrist C	18.02±0.86	17.28±0.86	0.001^**^

Definition: Finger Spans (FS1, FS2, FS3, FS4 and FS5), Finger Lengths (Thumb L, Index FL, Medial FL, Ring FL, Little FL), and 5 Perimeters (P1, P2, P3, P4, P5), Hand Shape: hand length / hand width, MF length; middle finger length, Forearm C; forearm circumference, Wrist C: Wrist circumference

**Table 3:** Relationship between handgrip strength and baseline characteristics of athletes and non-athletes

Hand Variable	Athletes (n=40)	Non-athletes (n=40)
Height (cm)	0.603(0.001^**^)	0.230(0.153)
Body mass (kg)	0.516(0.001^*^)	0.420(0.007^*^)
Lean body mass (kg)	0.536(0.001^**^)	0.469(0.002^*^)
Body fat (%)	0.0.79(0.630)	0.129(0.428)
BMI(kg x m^−1^)	0.061(0.704)	0.360(0.22)
Handgrip strength		
ND (kg)	0.850(0.001^**^)	0.713(0.001^**^)

* .α<0.05,

** .α<0.001

**Table 4:** *Relationship between handgrip strength and hand - specific anthropometric parameters, before and after controlling of body mass by partial correlation*.

Variable	Athletes (n=40)		Non- athletes(40)	

	Before	after	Before	After
FS1	0.541(0.001)	0.388(0.013)	0.185(0.252)	0.062(0.709)
FS2	0.539(0.001)	0.400(0.011)	0.202(0.212)	0.094(0.570)
FS3	0.555(0.001)	0.385(0.014)	0.217(0.178)	0.113(0.494)
FS4	0.512(0.001)	0.341(0.031)	0.266(0.098)	0.113(0.494)
FS5	0.565(0.001)	0.428(0.006)	0.220(0.172)	0.129(0.434)
TL	0.504(0.001)	0.240(0.135)	0.355(0.025)	0.158(0.337)
IFL	0.582(0.001)	0.379(0.016)	0.373(0.018)	0.218(0.182)
MFL	0.556(0.001)	0.354(0.025)	0.332(0.36)	0.180(0.272)
RFL	0.576(0.001)	0.385(0.014)	0.278(0.83)	0.121(0.464)
LFL	0.517(0.001)	0.340(0.032)	0.334(0.035)	0.161(0.327)
P1	0.635(0.001)	0.451(0.004)	0.313(0.050)	0.143(0.386)
P2	0.615(0.001)	0.426(0.006)	0.315(0.048)	0.155(0.347)
P3	0.591(0.001)	0.410(0.009)	0.312(0.050)	0.189(0.250)
P4	0.558(0.001)	0.373(0.018)	0.323(0.042)	0.187(0.254)
P5	0.643(0.001)	0.526(0.001)	0.314(0.049)	0.165(0.316)
Hand shape	−0.225(0.156)	−0.029(0.858)	0.146(0.368)	0.008(0.964)
Hand length	0.564(0.001)	0.332(0.036)	0.253(0.115)	0.150(0.363)
Palm length	0.530(0.001)	0.282(0.078)	0.216(0.181)	0.226(0.166)
Palm width	0.550(0.001)	0.371(0.018)	0.450(0.004)	0.246(0.131)
MF length	0.511(0.001)	0.305(0.056)	0.181(0.263)	−0.27(0.872)
Forearm length	0.604(0.001)	0.405(0.010)	0.071(0.665)	−0.65(0.694)
Forearm C	0.445(0.004)	0.162(0.319)	0.557(0.001)	0.406(0.010)
Wrist C	0.625(0.001)	0.468(0.002)	0.463(0.003)	0.246(0.131)

Definition: Finger Spans (FS1, FS2, FS3, FS4 and FS5), Finger Lengths (Thumb L, Index FL, Medial FL, Ring FL, Little FL), and 5 Perimeters (P1, P2, P3, P4, P5), Hand Shape: hand length / hand width, MF length; middle finger length, Forearm C; forearm circumference, Wrist C: Wrist circumference

**Table 5:** Stepwise multiple regressions in which handgrip strength was the dependent variable and finger span, finger length, or finger perimeters were the independent variables.

Group	Independent variable	R2×100	F	P
Finger span				
athletes	Fs5	30.20	18.29	0.001
Non-athletes	------	---	----	----
Finger length				
Athletes	IFL	32.20	19.98	0.001
Non-athletes	IFL	11.70	6.150	0.018
Perimeters				
Athletes	P5	39.80	27.48	0.001
Non-athletes	P4	8.10	4.42	0.042
